# Development of a Conceptual Model and Survey Instrument to Measure Conscientious Objection to Abortion Provision

**DOI:** 10.1371/journal.pone.0164368

**Published:** 2016-10-13

**Authors:** Laura Florence Harris, John Koku Awoonor-Williams, Caitlin Gerdts, Laura Gil Urbano, Ana Cristina González Vélez, Jodi Halpern, Ndola Prata, Peter Baffoe

**Affiliations:** 1 UC Berkeley–UCSF Joint Medical Program, School of Public Health, Berkeley, CA, United States of America; 2 Global Doctors for Choice/Ghana, Bolgatanga, Upper East Region, Ghana; 3 Ibis Reproductive Health, Oakland, CA, United States of America; 4 Global Doctors for Choice/Colombia, Bogota, Colombia; 5 Bixby Center for Population Health and Sustainability, School of Public Health, University of California, Berkeley, CA, United States of America; NHS Lothian and University of Edinburgh, UNITED KINGDOM

## Abstract

**Background and Objective:**

Conscientious objection to abortion, clinicians’ refusal to perform legal abortions because of their religious or moral beliefs, has been the subject of increasing debate among bioethicists, policymakers, and public health advocates in recent years. Conscientious objection policies are intended to balance reproductive rights and clinicians’ beliefs. However, in practice, clinician objection can act as a barrier to abortion access–impinging on reproductive rights, and increasing unsafe abortion and related morbidity and mortality. There is little information about conscientious objection from a medical or public health perspective. A quantitative instrument is needed to assess prevalence of conscientious objection and to provide insight on its practice. This paper describes the development of a survey instrument to measure conscientious objection to abortion provision.

**Methods:**

A literature review, and in-depth formative interviews with stakeholders in Colombia were used to develop a conceptual model of conscientious objection. This model led to the development of a survey, which was piloted, and then administered, in Ghana.

**Results:**

The model posits three domains of conscientious objection that form the basis for the survey instrument: 1) beliefs about abortion and conscientious objection; 2) actions related to conscientious objection and abortion; and 3) self-identification as a conscientious objector.

**Conclusions:**

The instrument is intended to be used to assess prevalence among clinicians trained to provide abortions, and to gain insight on how conscientious objection is practiced in a variety of settings. Its results can inform more effective and appropriate strategies to regulate conscientious objection.

## Introduction

Conscientious objection to abortion (CO) is defined as a clinician’s refusal to perform legal abortions because of religious or moral beliefs. Conscience-based objection to military service has been practiced since at least the Middle Ages, and conscientious objection in healthcare has emerged in recent decades, despite significant differences between military service and clinical care [1]. Abortion is a particularly common and contentious site of conscientious objection in healthcare, and the practice has generated increasing debate about how religious freedom intersects with abortion access [1–6]. CO is regulated through various policies, jurisprudence and guidelines around the world [7,8], although regulations have been criticized variously as inadequately protecting abortion access and inadequately addressing providers’ consciences [7,9–11]. Most CO policies require that conscientious objectors counsel patients on all pregnancy options including abortion, and refer them to willing providers [7]. Policies differ on whether health facilities can be exempted from providing abortions via claims of conscience, and on other aspects of conscience-based objection.

The scope and practice of conscientious objection have important consequences. The recent white paper on CO-related medical and public health literature by Global Doctors for Choice described how CO affects clinicians, patients, and health systems [7]. In particular, when CO acts as a barrier to abortion provision, it can contribute to morbidity and mortality from delayed or unsafe abortion [7,12]. Moreover, CO is the only sanctioned avenue for providers to refuse healthcare that would normally fall within their scope of practice. It is thus critically important to assess the prevalence of CO and to understand more about how CO works in practice.

In contrast to the substantial bioethical and policy literature about CO, relatively little is known about the issue from a public health perspective. The handful of studies on prevalence that exist globally have found that between 14% and 80% of clinicians refuse to provide contraception or legal abortions [7,10,13–15]. Qualitative work has revealed complexity and variation in how clinicians understand and practice CO, including lack of clinician knowledge about abortion and CO laws, lack of clear protocols at an institutional level, and clinician deviation from CO policies [9,16–18]. Moreover, there are rarely sanctions for providers who deviate from the legal framework for objection [19].

Global Doctors for Choice (GDC) is an international network of physician-activists, with action centers in Brazil, Colombia, Ghana, Mexico, and South Africa. GDC physicians called for more research on CO as it became an issue of increasing concern in the action center countries. A small number of quantitative studies have assessed prevalence of self-identified objectors—conscientious or otherwise—as one component of a larger survey (see Chavkin 2013 for a review). However, to our knowledge no quantitative survey instrument exists that focuses specifically on CO. There is a need for a quantitative instrument that assesses CO in clinical practice, because the practice and understanding of CO have implications for how it might be regulated.

To this end, an instrument was developed to measure the prevalence of CO and to give insight into its practice. The instrument can furnish data to inform effective and acceptable regulation of CO, and increase understanding of objection. It was designed for use in a variety of contexts, and was initially administered in Ghana by Global Doctors for Choice. This paper describes the conceptualization and development of the survey instrument and discusses the strengths and limitations of the instrument.

## Methods

**Fig 1:**
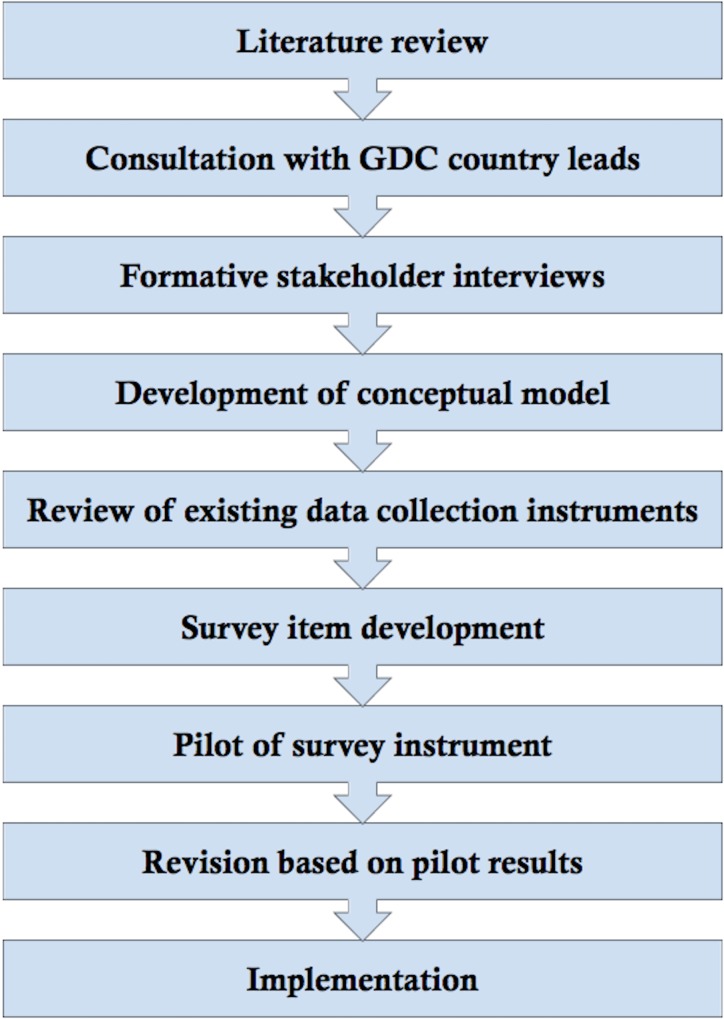
Process of survey development.

See Fig 1 for a summary of methods.

### Literature review

The literature review process was conducted from November 2013 to May 2015. PubMed, Google scholar, and Hein Online were used to search for published scholarship and opinion pieces in public health, ethics, and law. Professional networks of the authors were used to cull unpublished work and gray literature (e.g. conference proceedings). Literature in English, Spanish, French and Portuguese was included. The subjects reviewed included bioethical, policy, public health, and clinical aspects of CO, as well as relevant literature on related subjects, such as abortion stigma and the social and political context of abortion provision.

### GDC lead and co-lead physician consultation

LFH spoke with GDC lead and co-lead physicians from four of the five GDC country action centers (Columbia, Ghana, Mexico and South Africa) between February and May 2014; physicians from the Brazil action center were not available for consultation during this period. Discussions included the content areas of CO research that the lead and co-lead physicians thought were most important, how research could relate to their advocacy efforts, and their methodological and logistical considerations in conducting this research. These conversations and the literature review were used to design stakeholder interview guides.

### Stakeholder interviews

LFH conducted semi-structured, in-depth interviews with key stakeholders in Colombia in June and July 2014. Although Ghana was the site for pilot interviews and the first site for the survey administration, Colombia was chosen for initial interviews for several reasons. GDC has an action center in the country, and is interested in administering the survey in Colombia as well. Additionally, although Colombia has a lower maternal mortality rate and more elaborated CO policies than Ghana, the context in the two countries is similar in important ways. In both, abortion is legal for rape or incest, fetal abnormality, and physical or mental health of the woman [20,21]. Abortion access in both countries has expanded recently—Colombia legalized abortion in 2006, and Ghana adopted comprehensive abortion provision guidelines that same year which significantly increased access. Thus, both countries are still transitioning to more liberalized abortion access, and most clinicians in these countries underwent their pre-clinical training and early clinical practice when access was more restricted. In both countries, CO is a limited right; objecting clinicians must counsel patients on all options and refer to a willing provider [8,20,21]. Unsafe abortion is common in both, and abortion is stigmatized in both. Thus, it was considered that findings from Colombia interviews could provide a reasonable starting point for developing a tool that would be useful in Ghana as well–and ultimately in a variety of contexts.

Stakeholders in Colombia included conscientious objectors, abortion providers, psychiatrists, activists, health administrators, and legal experts. Psychiatrists were included because OB/GYNs in Colombia sometimes request that psychiatrists confirm patients’ claims of seeking abortion for mental health reasons. Responses were recruited via email from the professional networks of GDC/Colombia country lead and co-lead physicians, and were purposively selected to ensure variation in profession, place of work, and known opinions about CO. Interviews were conducted in Spanish or English depending on respondent preference. Interviews conducted in Spanish were facilitated by a Spanish language interpreter. The interviews were audio recorded and transcribed verbatim. Transcripts were analyzed in NVivo by LFH using thematic analysis. Initial themes were derived from the transcripts, discussed with authors, and revised accordingly. All interviews undertaken as part of this research received IRB approval from UC Berkeley (CPHS # 2014-03-6178). The IRB waived the need for written consent from respondents. All respondents gave verbal informed consent prior to being interviewed, consistent with protocol. Respondents were not compensated. Interview guides for formative and pilot interviews available on request.

### Development of conceptual model

A conceptual model of conscientious objection was formulated from the themes of the interviews and literature review. Domains for the survey were based on the conceptual model as well as themes from the interviews and literature review.

### Review of existing data collection instruments

Survey instruments and interview guides from related fields and topics of study were collected for review. Research tools included instruments or excerpts thereof that accompanied published articles, instruments that were publicly available online, and unpublished instruments and guides used in both published and unpublished research (collected with authors’ permission). Instruments were in English, Spanish, French, and Portuguese. Of the 14 instruments collected, six were close-ended survey instruments [4,22–26], and eight were open-ended, in-depth interview guides [27–31] (personal communication with Joanna Mishtal and Bethany Kotlar).

The citations given are for tools that are publicly available, or for published work analyzing data gathered by the tools. The tools were entered into a database for comparison, with emphasis placed on identifying phrasing that could be used for the present survey.

### Survey item development

Items were developed within the survey domains based on interview and literature review findings. When possible, questions from other surveys were used as written or in adapted form, to maximize questions that had already been field-tested and validated, and to provide opportunities for comparisons between survey instruments. The survey was tested for basic comprehension and subjective impression of validity with colleagues from the Upper East Regional Health Bureau in Ghana, who suggested some wording changes.

### Pilot testing of survey instrument

The survey was pilot tested in January 2015 with doctors and midwives who were currently practicing in hospitals in three regions in northern Ghana. Pilot respondents were recruited in person from hospitals in the Upper East and Northern regions of Ghana, both of which were part of the planned study area for administration of the finalized survey. Respondents were purposively selected for variation between clinician type (physicians or midwives) and ownership of health facility where employed (public, private, or Christian Hospital Association of Ghana). In Ghana, some midwives have received training in comprehensive abortion care (CAC); others have received more limited abortion training through other programs, might participate more peripherally in abortion services, or might not have received any training. Both CAC-trained and non-CAC-trained midwives were interviewed. Respondents completed the survey instrument via one of three methods: self-administration using a tablet, self-administration using paper, and administration via interview by LFH. After completing the survey, LFH asked respondents open-ended questions about their thoughts on the instrument’s content and phrasing. These post-survey interviews were audio-recorded and transcribed. Transcripts were analyzed in NVivo using thematic analysis, and were triangulated with survey instrument responses. Themes were discussed with the authors, and revised accordingly. All respondents gave verbal informed consent prior to participation. Respondents were not compensated.

### Post-pilot revision

The survey instrument was modified based on results from the pilot study. A team of experts reviewed this modified instrument; their feedback was incorporated. As a pre-test, the modified survey was administered to a midwife who was working at a hospital in the survey area, to check comprehension of items. Small modifications were made to instrument wording based on the results of this pre-test.

See Fig 1 for an overview of the methods used to develop this survey instrument. All interviews were conducted and analyzed by LFH.

## Results

### Literature review

The literature review highlighted the potential for differences between CO policies and its practice [32]. Specifically, the review found that CO policies were often unclear, leaving key issues open to interpretation [11,18]. There was evidence of poor knowledge and/or understanding of CO and abortion policies among clinicians and health administrators [33]. Further, the review found that contextual pressures can affect the practice and understanding of CO. High workload, low pay and disapproval from health administrators can discourage abortion provision [18]. As the only legal way to refuse to provide abortions, CO can become a safety valve for clinicians under pressure and may be used by clinicians who do not have moral or religious objections. Social factors including stigma also shape the ways that stakeholders and policymakers approach CO [4]. Due to the above issues, in many countries CO policies do not appear to achieve their intended effect of protecting clinicians’ consciences while also maintaining abortion access [7,10]. However, in some countries conscientious objection achieves these goals to a significant extent. For example, in Portugal and Norway the national health systems must ensure abortion provision by paying specific providers, and in Great Britain, independent subcontracted organizations provide a significant proportion of abortions.

### Stakeholder interviews

Eleven interviews were conducted with a total of 13 respondents. In two cases, the interviews were conducted with two respondents at the same time; the paired respondents were colleagues in both cases. The 13 respondents consisted of three conscientious objectors (two OB/GYNs, one generalist), three abortion providers (OB/GYNs), two psychiatrists, two public health researchers, a constitutional court expert, a health administrator, and a reproductive rights activist. Interviews lasted between 30 and 80 minutes.

#### Regulation vs. practice of CO

Respondents thought that Colombia’s CO-related jurisprudence had created a fairly strong and clear legal framework, but that CO practices varied by clinician and institution, and often differed from the practices mandated by court decisions. Several respondents discussed how laws did not translate into clear clinical regulations.

*“Colombia is a country of laws*, *but they are not enforced*,*”* (OB/GYN who provides abortions 1 [number distinguishes between respondents with the same professional role]).

*“None of this is regulated*, *so then we know that in practice*, *nothing happens*. *The doctor simply says to the patient ‘I am an objector*, *so look for someone else*.*’ It doesn’t happen as it should*,*”* (health administrator 1).

Several respondents mentioned that an unsupportive regulatory environment helped to enable deviations between CO in law and in practice. For example, the “*procurador”* is a civil office charged with ensuring public servants’ compliance with the law (including health professionals). The current *procurador* is anti-choice:

“[*The procurador’s] personal agenda intersects with his public responsibility… providers are afraid to act*, *because of course*, *they are monitored by an entity that does not agree with this issue [of abortion]*,*”* (reproductive rights activist 1).

Interviewees characterized the practice of CO as affected by factors ranging from health administration policies to stigma about abortion provision, and stated that it was difficult to separate CO from this context.

#### Beliefs related to CO

Respondents thought that beliefs about abortion, and motivations for refusal, were of central importance to CO. They described that stigmatizing or paternalistic beliefs could fuel clinicians’ refusal to provide abortions, and all but one thought that these beliefs should be distinguished from conscience-based beliefs.

*“It is easier to say no than to say yes*. *When you say yes*, *you are committing to many things*. *When you say no*, *you shut a door*, *and there*, *no one will bother you*,*”* (generalist who identifies as conscientious objector 1).

Lack of respect for or knowledge about human rights was also thought to contribute to improper CO.

*“Many who consider themselves conscientious objectors don’t know the policy*, *don’t know the law*, *don’t know practices about abortion…and above all*, *don’t know about women’s rights*,*”* (OB/GYN who provides abortions 2).

#### Actions

Respondents discussed how some objectors would not counsel or refer patients appropriately, or would create unnecessary administrative tasks. For respondents, these actions de-legitimized clinicians’ claims of conscientious objection.

*“They [some physicians] try to hinder women from obtaining abortion*. *And they put up barriers and barriers and barriers*. *That to me is no conscientious objection*. *I think some of them consider themselves conscientious objectors*, *because of the simple fact that they think it is wrong to interrupt a pregnancy*,*”* (health administrator 1).

One respondent, a conscientious objector, stated that he was respectful of reproductive rights but that he would try to dissuade patients from obtaining abortions.

*“Objecting doctors should simply ask the patient if she desires [an abortion] or not*, *and then send her to doctors who will do it*, *but in my case I try to dissuade them a bit because it shouldn’t be done*, *in my religion*,*”* (OB/GYN who identifies as a conscientious objector 2).

The other conscientious objectors did not comment on counseling.

#### Self-identification

Most respondents thought that CO meant different things to different clinicians, and that the concept was unclear for many.

*“In reality*, *many who think they are conscientious objectors are unaware of the laws*, *unaware of abortion practices*, *unaware of women’s rights*, *especially*. *When we talk to them and tell them what [CO] is*, *many of them understand that they are not conscientious objectors really*, *they simply don’t know the law*,*”* (OB/GYN who provides abortions 1).

As described above, respondents spoke about how clinicians might call themselves conscientious objectors, but act as obstructers–for example, by not making a referral or by setting up unnecessary administrative hassles. These sorts of clinicians, who neglect some or all of the responsibilities that come with conscientious objection such as counseling and referring, cannot be considered proper conscientious objectors according to Colombian regulations and jurisprudence. Additionally, respondents described how some clinicians call themselves conscientious objectors even though they aren’t eligible to object. For example, although Colombian policy states that only those who can perform abortions are able to claim conscientious objector status:

*“Starting with administrative levels there are those who claim status as conscientious objectors*, *be they secretary*, *receptionist*, *or gatekeeper*. *Those levels of barriers arise long before medical contact*,*”* (health administrator 1).

Some interviewees stated that clinicians might resist categorizing themselves definitively.

*“Nobody wants to be identified as a non-objector or objector or because in the morning you can work in a religious institution*, *and in the afternoon [in a non-religious institution]…it changes*,*”* (psychiatrist 1).

The above quotation also highlights the ways that clinicians’ decisions about abortion provision may be influenced or determined by context.

### Conceptual model of CO

**Fig 2:**
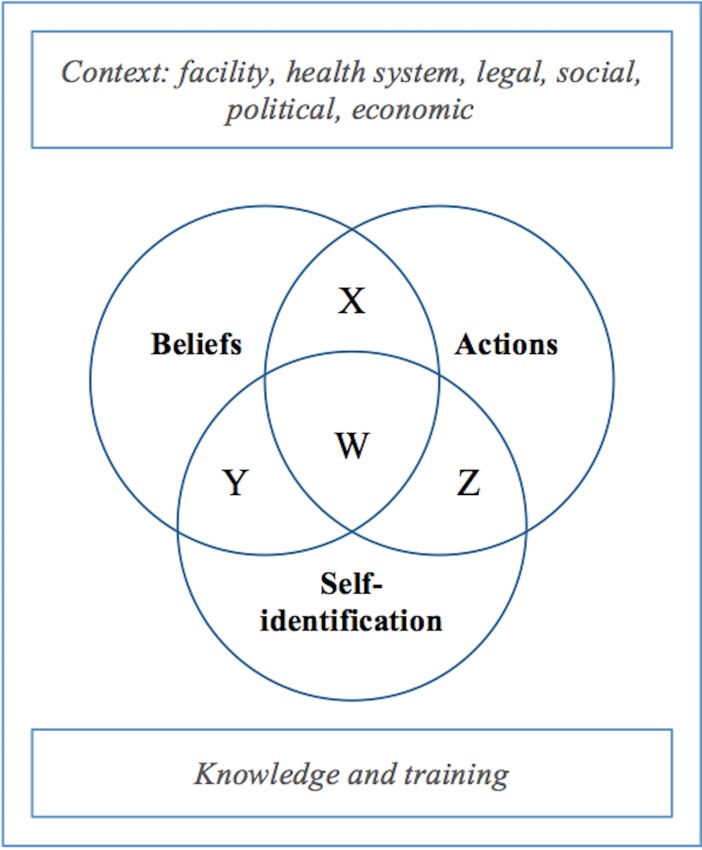
Domains of Conscientious Objection. The three principal domains are actions, beliefs, and self-identification.

Based on the literature review and stakeholder interviews, a conceptual model of CO was developed. The model posits three domains of CO: *beliefs*, *actions*, *and self-identification* (Fig 2). The *actions* domain includes whether a clinician provides abortions, and whether he or she counsels and refers patients appropriately. If the clinician doesn’t provide abortions but counsels and refers appropriately, she would be in the actions circle because her actions correspond with CO policy. Clinicians who provide abortions, or who don’t provide but also don’t refer, would be outside the circle, because they don’t follow the actions of a conscientious objector according to policy. The *beliefs* domain includes whether a clinician is morally opposed to abortion, and related beliefs about abortion stigma and reproductive rights. Clinicians who are morally opposed to abortion would fall within the beliefs circle, consistent with the policy definition of CO. The *self-identification* domain is whether a clinician calls him or herself a conscientious objector; self-identified objectors fall within the self-identification circle. These three domains contain some objective (verifiable) criteria and some subjective (non-verifiable) criteria.

Most conscientious objection policies, as well as most bioethicists, define a proper conscientious objector as a clinician who does not provide abortions based on moral or religious beliefs, but who counsels and refers [1,7]. This definition implicitly assumes that the three domains align in individuals who object (W, on Fig 2). However, the domains may not align in practice, or even in some clinicians’ understanding of the concept. A clinician may believe that abortions are morally wrong and refuse to provide them, but not identify as a conscientious objector because she is unfamiliar with this concept, or it is not how she thinks about herself (X). Alternatively, a clinician might identify as a conscientious objector and believe that providing abortions is a sin, but if he does not refer appropriately he would be considered an obstructer of abortion access rather than a conscientious objector (Y). An obstructer refuses to provide abortions but does not fulfill the responsibilities of a conscientious objector such as counseling or referral. All three domains of beliefs, actions, and self-identification contribute to the understanding and practice of CO, but each domain has different implications for regulation.

Prerequisites for the provision of abortion–or meaningful objection to it–are adequate training to provide abortions, and adequate knowledge of abortion and CO laws. Thus, assessing training and knowledge is important to understanding prevalence. Institutional context also affects CO, and should be considered as part of comprehensive research about CO. Assessing clinician views about potential regulations of CO can also directly inform policy strategies.

### Creation of survey items

The survey sections were based on the domains of conscientious objection as identified by the conceptual model (self-identification, actions, beliefs). Sections on demographics, training, and policy opinions were also included in the instrument. Within all sections, survey items were created based on the interview findings and literature review results. As an example of survey item creation, take one interviewee’s observation that *“Many who consider themselves conscientious objectors don’t know the policy*, *don’t know the law*, *don’t know practices about abortion…and above all*, *don’t know about women’s rights”*. Based on this observation and similar observations made by other interviewees, the survey instrument includes items that assess the respondents’ knowledge of CO policy in the country of administration, items about their training in abortion and comfort with that training, and an item that assesses agreement/disagreement with the statement “Every woman has the right to access safe abortion to the full extent of the law.” (See appendix for the full survey instrument.)

Items from previously field-tested and validated survey instruments were used when possible; these represented a minority of all survey items. Items were taken from three close-ended surveys: the Ghana Health Providers Survey component of an evaluation of the program Reducing Maternal Mortality and Morbidity (R3M) [24], Stigmatizing Attitudes, Beliefs and Actions Scale (SABAS) [23], and the abortion provider stigma survey (APSS) [25]. The survey instrument is presented as supplementary material (S1 File).

### Pilot of survey instrument

The nine respondents consisted of two doctors and seven midwives who were offering sexual and reproductive health services at hospitals in Northern Ghana—specifically, in the planned study area where the finalized survey instrument would be administered on a large scale. Few doctors were interviewed because of the small number of doctors in the recruitment region which would eventually be used for survey administration. Three of the seven midwives were CAC-trained. Practitioners worked at five hospitals: one MD and one midwife worked at a Christian hospital; one midwife worked at a private hospital, and the other respondents worked at three different public hospitals. Four of the nine providers did not want the post-survey interview to be audio-recorded; notes were taken about these interviews, with providers’ permission. Interviews lasted between 15 and 30 minutes, with additional conversation occurring during survey tool administration.

Overall, respondents said that the domains and survey items were relevant to the concept of CO, although most said that the survey was too long. The subsequent themes are those that differed from the stakeholder interviews, and that added nuance to the conceptual model of CO.

#### Framing of abortion as safe/unsafe, rather than legal/illegal

Most interviewees lacked clarity about abortion law and CO policies, but all had vivid understandings of the health consequences of unsafe abortions. Thus, their primary focus in terms of counseling, provision, and referral was not whether the abortion was legal–or even moral–but whether the patient was likely to seek an unsafe abortion.

*“[Whether abortion is provided] depends on the reason they give*. *If it is convincing enough then you do it and if you think that if you don’t do it the person can end up doing something unsafe to cause her death then you do it for them*,*”* (midwife who provides abortions 1).

*“When you have a law that is so loose such that you can always find a reason around the law to carry out the process*, *then it looks like there is literally no law*. *And you have people carrying out the procedure in so many places and they come in here complicated*. *Personally*, *my reason for carrying out abortion will be if I don’t do it for her she is going out to see a quack who will have it done*,*”* (doctor who provides abortions 1).

#### Counseling vs. provision

According to most CO policies, including the policy in Ghana, a conscientious objector is expected to exercise his or her conscience through refusing to perform abortions, but must provide unbiased counseling. However, some interviewees’ expression of conscience was through biased counseling rather than provision: they would provide abortions that they considered immoral if they were worried about the patient seeking unsafe abortion, but would do their best to dissuade patients who they thought were unsure about their decision.

#### Moral beliefs and stigma

Most respondents were highly religious, and most provided abortions. These clinicians reported that their religion looked negatively on abortions, but separated their religious and professional identity.

*“Religion can’t taboo [abortion provision]–it’s my work*,*”* (midwife who provides abortions 2).

However, some interviewees’ moral beliefs seem highly connected with stigma. One interviewee who indicated via the survey that religious/moral beliefs were the reason she did not perform abortions for some indications, clarified during the interview that sometimes she wouldn’t refer patients as “punishment”—for example, because those patients should have known to use contraception. When this clinician referred to moral beliefs, she wasn’t speaking of beliefs about her personal involvement in providing the abortion, but a moral judgment of patients. It appeared that stigma interacted with moral beliefs by shifting the moral calculus—i.e. it would not be right to provide an abortion for a certain patient because the pregnancy was ‘her fault’.

#### Clinician reactions to the survey instrument

Most respondents said that they had learned new things from the survey or that it had refocused their thinking about CO, counseling, and abortion.

*“I think [the survey] is a good thing because we haven’t actually thought about that for a long time as to whether or not the person believes in [providing abortions]*, *though some object when they come but we haven’t really taken it so serious*,*”* (midwife who provides abortions 3).

Some respondents said that the survey would not change the way they practiced; others that it would. Respondents said that they would like to receive information about CO and abortion law after taking the survey.

## Measuring Objector Status

Based on the conceptual model and survey instrument development process, items from the instrument can be combined to determine conscientious objector status for each respondent. According to the conceptual model—and indeed, most CO regulations—providers responding to the survey can be classified as a conscientious objector if they have been trained to provide abortions, and meet all of the following criteria:

1. Respond to at least one hypothetical abortion scenario by indicating that they would not provide the abortion (in some countries, this may mean responding to all)
2. Indicate that they would counsel and refer patients in scenarios in which they do not provide abortions
3. Indicate religious or moral belief as a reason for lack of provision
4. Indicate that they self-identify as a conscientious objector

Prevalence of legitimate objection can be calculated as the percentage of providers who meet all four criteria.

Related populations of interest may be useful for comparison, such as “self-identified conscientious objectors” (those who meet criterion D without meeting one or more other criteria), “illegitimate objectors” (those who meet criterion A but fail to meet one or more other criteria). The information gathered from the survey can also allow exploration of nuances such as how to classify and make sense of the status of clinicians who are working in institutions that do not provide abortions.

## Discussion

The literature review, interview, and pilot results were used to develop and refine a conceptual model of CO. A survey instrument was created to reflect the three domains of CO: belief, action, and self-identification; it can be used to measure the prevalence of CO and to obtain information about its practice. The instrument is not intended to be analyzed as a scale; the three domains of CO should each be considered in their own right. The instrument also includes sections on training, knowledge about CO and abortion law, and on opinions about potential policies to regulate CO. Data obtained through this instrument can be used to deepen understanding of the practice of CO; to inform policy, advocacy and public health strategies; and to understand how clinicians might respond to regulations around CO.

### Nuanced and varied rationales for CO

The simple definition of CO–objection that stems from moral, religious or ethical beliefs–belies the complex sets of factors that clinicians consider when deciding whether they identify as conscientious objectors. Interview findings suggest nuance and variation in how clinicians conceptualize moral beliefs and relate them to medical practice. The extent to which morality is intertwined with stigma and judgment is particularly striking. Further, CO is derived from a Western bioethical framework, and is usually understood as a balance between individual liberties: of the clinician, and of the woman to determine her reproductive fate (1). While this framing is implicit in the policies and guidelines around the world–for example, as the rationale behind the need for conscientious objectors to counsel appropriately and refer–non-Western clinicians may not share this lens. For example, some respondents valued abortion primarily as a means to prevent maternal mortality rather than a means to ensure reproductive rights. Additionally, some respondents thought of medical work as entirely separate from their personal moral or religious belief system. The survey includes items about the respondents’ perspective on stigma, reproductive rights, and similar issues, in an effort to probe for some of these nuances.

Despite the variation in conceptualizations of CO, we believe that the instrument provides a reasonable way to measure its prevalence, and that it is far more meaningful than measurement self-reported objector status, which is the basis for most existing data on prevalence. There are still many issues that remain in conceptualizing CO, and although this survey provides what we consider to be an adequate working definition based on our knowledge and understanding of CO at this time, the quantitative measurement of CO can likely be iteratively improved as we understand more about this phenomenon.

### Qualitative work and validation

Quantitative data is useful in understanding the magnitude of CO and major trends in the way that it is practiced. However, given the complexity of CO, this survey should be paired with qualitative research to lend further, more nuanced understanding of results. Qualitative work can help validate findings and suggest improvements for future iterations of the survey, and can give insights on the conceptual model’s strengths and limitations. More insight will be gained about this survey instrument once the results from the first implementation in Ghana have been analyzed, and complementary qualitative work will be done in the study area to contextualize the quantitative findings. Further work should be done to validate this survey, and to investigate the process of adaptation for other settings.

However, validation is challenging because the very concept of CO is still under-theorized. For example, one strategy for validation could be to pair this survey with the full SABAS (Stigmatizing Attitudes, Beliefs and Actions Scale) and assess overlap. However, a high degree of overlap could be taken to suggest that the CO instrument is mistakenly assessing abortion stigma instead of “true” CO, or could be taken to indicate that stigmatizing beliefs and CO do indeed have a great deal of overlap, as was discussed in several interviews.

### Administration of instrument in other settings

The survey was developed through an internationally focused literature review and interviews in Colombia and Ghana. As described in the methods section, the two countries were chosen in part because of their similarities in abortion and CO policy and practice, and partly because of their differences, in an attempt to create an instrument that would be applicable in a variety of contexts. A particularly important similarity is that Colombia and Ghana had recently made abortion legal or increased access via health system guidelines, respectively, and were thus undergoing a transition period. This may limit the extrapolation of results to countries with stable abortion policies.

Nonetheless, the basic domains of the conceptual model–actions, beliefs, and self-identification–were relevant in both Colombia and Ghana, and it seems reasonable that they would be relevant in most contexts. As shown from the differences in interviews between the two countries despite their contextual similarities, the domains may look different in different contexts. Survey items will likely need to be adapted accordingly. We recommend that adaptation be based on qualitative research in the local context if possible, or at least with the input of local stakeholders familiar with local policy and practice.

### Pedagogical implications

The survey may function as a values clarification exercise for some respondents. If most or all clinicians on a unit take the survey at the same time, it presents an opportunity to start a unit-level conversation about CO, based on the survey material. However, taking advantage of pedagogical opportunities may change the phenomenon that the survey measures; as clinicians gain understanding of CO, they may change their minds about whether they identify as objectors. This instrument attempts to gain an estimate of CO from at least minimally informed clinicians, by providing the definition of conscientious objection. The survey instrument asks questions about CO and abortion policy; it can be followed with a fact sheet of country or region-specific answers to these questions in order to increase clinician knowledge, and ensure that they are informed of objectors’ obligations to counsel appropriately and provide referrals.

Additionally, the survey instrument could be used to measure changes in clinicians’ conscientious objector status, and related beliefs and practices, after a specific intervention such as a values clarification workshop.

### Political implications

Attention should also be given to the survey’s potential political implications. One concern might be that simply administering the survey might change respondents’ behavior–perhaps in ways that reduce abortion access. However, refusals to provide legal abortions happen in most contexts, and if clinicians are not aware of the right to conscientiously object then they are not aware of the limits of this right. We believe that the survey should be administered in the context of appropriate complementary education that underscores the importance of CO’s limited scope.

Further, by asking separately about different components of CO–and by asking respondents what CO regulations they deem acceptable–the survey can give insight into ways to shape CO-related policies and programs. For example, if clinicians who refuse to provide abortions do not identify as conscientious objectors, this may suggest the need for an informational campaign about CO. If clinicians who provide abortions do not counsel and refer, programming or policies to reinforce the limited scope of CO may be appropriate. Alternatively, if clinicians claim objector status because of fear that they will be stigmatized, rather than moral or religious beliefs about abortion itself, interventions to reduce abortion stigma may be key in addressing CO. Another option for regulation that is seen in a few countries with high public acceptance of abortion (Iceland, Finland, and Sweden) is to not allow conscientious objection at all. In these countries, clinicians who object to abortion provision mainly specialize in fields other than OBGYN or midwifery, where they are not expected to provide abortions [34].

### Limitations

A quantitative survey about CO is necessarily limited in several ways. Respondents may be reluctant to disclose ways that their practice deviates from CO policy because of social desirability bias; for example, some providers may identify themselves as conscientious objectors in some practice settings but not others. Additionally, objection can occur at many stages before abortion provision (e.g. through asking receptionists to not schedule appointments with people seeking abortion), and the instrument could not reasonably ask about all types of objections.

Regarding the development process, only one person (LFH) analyzed the interview data from all sets of interviews. However, there was feedback at key points from the authors and the GDC team working on the study. Another limitation was that the respondents in Colombia were all professional contacts of the GDC country lead physicians. This recruitment strategy meant that even the conscientious objectors were more likely to recognize the importance of reproductive rights, which limited the range of viewpoints considered in the study. During the pilot in Ghana, the same person administered surveys and conducted post-survey interviews. This may have increased social desirability bias from the respondents, leading them to give more favorable reviews of the survey and to be more reluctant to discuss aspects that they found lacking.

### Strengths of the development process

This is the first quantitative instrument of which we are aware that assesses CO in a robust manner. The survey benefited from feedback from a team at GDC and technical experts. There was breadth in the locations and perspectives considered in the literature review, interviews and pilot. Moreover, the instrument development process furnished a conceptual model of conscientious objection that may be useful for research beyond the specific survey instrument.

## Conclusions

Measurement of CO plays an important role in clarifying distinct aspects of this complex phenomenon. Moreover, it is a necessary part of formulating effective regulations that protect reproductive rights and clinicians’ beliefs. CO is under-theorized and under-researched. More qualitative and quantitative work is needed to understand how clinicians understand and practice CO.

## Supporting Information

S1 File Survey Instrument: Prevalence of conscientious objection to legal abortion among clinicians in Northern Ghana. (PDF)
