# Supplementary material for: Development of a Conceptual Model and Survey Instrument to Measure Conscientious Objection to Abortion Provision
Source: PLoS One. 2016 Oct 13;11(10):e0164368. doi: 10.1371/journal.pone.0164368 (PMC5063579; doi:10.1371/journal.pone.0164368)
Supplement: S1 File — (PDF) [file pone.0164368.s001.pdf]

Thank you for filling out this survey. We value your opinions and are interested in hearing what you think. There is no "right" answer for many of these questions, so please answer from your own knowledge and opinions to the best of your abilities. Moreover, please be assured that your responses are confidential.

Section 1: Demographics

1.01 Are you:
- ☐ Male
- ☐ Female

1.02 How old were you on your last birthday?______ (years)

1.03 What is your title at this hospital?
- ☐ Ob/Gyn
- ☐ Medical Officer
- ☐ Midwife, trained in Comprehensive Abortion Care
- ☐ Midwife trained on the job in abortion care
- ☐ Other (please specify) ________________

Section 2: Training and provision

2.01 Please mark whether you have been trained to perform the following types of abortion:

Type of abortion	Yes	No
Medication abortion (with misoprostol or mifepristone/misoprostol)	☐	☐
Manual and/or electric vacuum aspiration	☐	☐
Dilation and Curettage (D&C)	☐	☐

2.02 Up to what gestational age were you trained to provide abortions? ______ weeks

2.03 Have you ever been selected for an abortion-related training but not participated?
- ☐ Yes
- ☐ No →***skip to 2.05***

2.04 If you were selected for an abortion-related training but did not participate, why not? (**Check all that apply.)**
- ☐ Training time didn't fit with my schedule
- ☐ Providing abortions is against my moral or religious beliefs
- ☐ Fear of being stigmatized by my colleagues
- ☐ Fear of administrators/management' disapproval
- ☐ Fear of my family's/community's disapproval
- ☐ Not wanting to add another responsibility to my workload
- ☐ Not interested in providing abortion
- ☐ Other (please specify) ___________________________

2.05 Have you ever personally provided an abortion?
- ☐ Yes
- ☐ No→***skip to 2.07***

2.06 Do you personally currently provide any abortions in this hospital?
- ☐ Yes
- ☐ No

Please indicate what you personally would do in the following clinical scenarios:

Scenario 1 At your current job, you see a patient who is 17 years old, pregnant, and does not want to keep the pregnancy because she is worried that she will not be able to continue her studies if she has a baby.

2.07 Do you counsel her on all options regarding the pregnancy, including abortion?
- ☐ Yes
- ☐ No

2.08 Do you try to convince her to keep the pregnancy?
- ☐ Yes
- ☐ No

2.09 After counseling, this patient wants an abortion. Do you perform the abortion?
- ☐ Yes→ ***skip to 2.11***
- ☐ No
- ☐ Not sure

2.10 If no or not sure, which factors contribute to your response?

	Not at all	Somewhat	A great deal
a) My personal religious or moral beliefs about abortion	☐	☐	☐
b) The patient should have known better	☐	☐	☐
c) My hospital administration does not support abortions	☐	☐	☐
d) I am worried that my family or community would disapprove	☐	☐	☐
e) Other (specify)________________________	☐	☐	☐

2.11 If you do not provide the abortion, do you refer to a clinician who will provide one?
- ☐ Yes
- ☐ No
- ☐ Not sure

Scenario 2 You see another pregnant patient who is 33 years old, does not want to be pregnant and has pulmonary hypertension, which has a 50% mortality rate during pregnancy.

2.12 Do you counsel her on all options regarding the pregnancy, including abortion?
- ☐ Yes
- ☐ No

2.13 Do you try to convince her to keep the pregnancy?
- ☐ Yes
- ☐ No

2.14 After counseling, this patient wants an abortion. Do you perform the abortion?
- ☐ Yes→ ***skip to 2.17***
- ☐ No
- ☐ Not sure

2.15 If no or not sure, which factors contribute to your response??

	Not at all	Somewhat	A great deal
a) My personal religious or moral beliefs about abortion	☐	☐	☐
b) The patient should have known better	☐	☐	☐
c) My hospital administration does not support abortions	☐	☐	☐
d) I am worried that my family or community would disapprove	☐	☐	☐
e) Other (specify)________________________	☐	☐	☐

2.16 If you do not provide the abortion, do you refer to a clinician who will provide one?
- ☐ Yes
- ☐ No
- ☐ Not sure

Scenario 3: You see another patient, a 25-year-old woman who has become pregnant as the result of rape.

2.12 Do you counsel her on all options regarding the pregnancy, including abortion?
- ☐ Yes
- ☐ No

2.13 Do you try to convince her to keep the pregnancy?
- ☐ Yes
- ☐ No

2.14 After counseling, this patient wants an abortion. Do you perform the abortion?
- ☐ Yes→***skip to 2.17***
- ☐ No
- ☐ Not sure

2.15 If no or not sure, which factors contribute to your response??

	Not at all	Somewhat	A great deal
a) My personal religious or moral beliefs about abortion	☐	☐	☐
b) The patient did not provide evidence for rape	☐	☐	☐
c) My hospital administration does not support abortions	☐	☐	☐
d) I am worried that my family or community would disapprove	☐	☐	☐
e) Other (specify)________________________	☐	☐	☐

2.16 If you do not provide the abortion, do you refer to a clinician who will provide one?
- ☐ Yes
- ☐ No
- ☐ Not sure

2.17 Do you know of a clinician to whom you can refer patients for abortion?
- ☐ Yes, in this facility
- ☐ Yes, in another facility
- ☐ No

2.18 Until what gestational age do you perform abortions? ______ weeks

2.19 Why is this the latest gestational age you perform abortions?
- ☐ I was only trained to provide abortion services up to this gestation
- ☐ It is against my moral or religious beliefs to provide abortion past this gestation
- ☐ Other (specify)________________________________

2.20 Have you ever advised a woman to take misoprostol and then seek postabortion care?
- ☐ Yes
- ☐ No

2.21 Have you ever refused to provide abortions at one facility, but provided them in another?
- ☐ Yes
- ☐ No

Section 3: Perspectives

3.01 Are you uncertain about the circumstances under which you can legally provide abortion?
- ☐ Yes
- ☐ No

3.02 Are you uncertain about how to perform a safe abortion?
- ☐ Yes
- ☐ No

3.03 Please indicate whether you personally disagree with, have mixed feelings about, or agree with the following statements.

	**Disagree**	**Mixed feelings**	**Agree**
a. The needs of a patient are more important than the beliefs of a clinician.	☐	☐	☐
b. Clinicians have a responsibility to counsel patients against having an abortion.	☐	☐	☐
c. Every woman has the right to access safe abortion to the full extent of the law.	☐	☐	☐
d. Providing abortions is a positive contribution to society.	☐	☐	☐
e. I feel that providing abortions is morally wrong.	☐	☐	☐
f. I feel guilty about providing abortions.	☐	☐	☐
g. I do/would worry about telling people that I provide abortions.	☐	☐	☐
h. A woman who has had an abortion brings shame to her family.	☐	☐	☐
i. A woman who has an abortion is committing a sin.	☐	☐	☐
j. The later the gestational age, the more sinful the abortion.	☐	☐	☐
k. I would continue to be friends with someone if I found out that they had an abortion.	☐	☐	☐
l. Most abortions could be provided under the legal ground of "mental health".	☐	☐	☐

Section 4: Policy

4.01 Please mark whether abortion is legal or illegal in Ghana in the following cases:

In the case of…	Legal	Illegal	Don't know
Rape	☐	☐	☐
Incest	☐	☐	☐
Serious fetal malformations	☐	☐	☐
Risk to woman's life	☐	☐	☐
Mentally impaired woman	☐	☐	☐
Risk to psychological health of woman	☐	☐	☐
Risk to physical health of woman	☐	☐	☐
Socioeconomic grounds	☐	☐	☐
Under any circumstances	☐	☐	☐

4.02 Sometimes clinicians who are trained to provide abortions refuse to provide abortions because of their religion or their moral beliefs. Such refusal is called conscientious objection, because they are *objecting* to providing a medical service because of their *conscience.* Have you heard about conscientious objection?

- ☐ Yes – I have heard about the term
- ☐ Yes – I have heard about the idea, but not the term
- ☐ No – I have not heard about the idea or the term → ***skip to 4.04***

4.03 In what settings have you learned about conscientious objection to abortion provision? **(Please check all that apply)**

- ☐ Pre-service education
- ☐ In-service training session
- ☐ From supervisors/management
- ☐ From colleagues
- ☐ Religious organization (e.g. Church, Christian association, Mosque)
- ☐ Other (specify) ________________
- ☐ I have not received education about conscientious objection

4.04 The following statements present different aspects of conscientious objection. For each, please indicate whether or not the statement is currently true according to the Ghanaian Standards and Protocols, **and** whether or not you think it should be true. Remember we are interested in knowing your personal opinion.

	The case in Ghanaian Standards & Protocols?			Do you think it should be the case?	
**Aspect of conscientious objection**	**Yes**	**No**	**Don't know**	**Yes**	**No**
Clinicians who conscientiously object must counsel patients with unwanted pregnancies on all of their treatment options, including abortion.	☐	☐	☐	☐	☐
Clinicians who conscientiously object must refer patients eligible for a legal abortion to a clinician willing to provide it.	☐	☐	☐	☐	☐
Only a clinician who would be performing the abortion is eligible to conscientiously object – i.e. secretaries, assistants cannot conscientiously object.	☐	☐	☐	☐	☐
Clinicians can be conscientious objectors to postabortion care.	☐	☐	☐	☐	☐

4.05 Do you consider yourself a conscientious objector to abortion provision (i.e. someone who refuses to provide abortions based on personal moral or religious beliefs?)?

- ☐ Yes
- ☐ No

4.06 Do you fit the definition of a conscientious objector, according to Ghanaian policy?

- ☐ Yes
- ☐ No
- ☐ Unsure

4.07 Do you conscientiously object to taking care of a woman with complications after an abortion (i.e. postabortion care)?

- ☐ Yes
- ☐ No

Section 5: The workplace

5.01 Are abortions ever performed at this hospital?

- ☐ Yes
- ☐ No

5.02 Does this hospital have a formal policy prohibiting abortion, due to moral or religious grounds?

- ☐ Yes
- ☐ No

5.03 Does this hospital have a formal policy about conscientious objection (i.e. a policy about the rights and responsibilities of trained clinicians who refuse to provide abortions because of moral or religious beliefs)?

- ☐ Yes (specify the policy) ____
- ☐ No
- ☐ Don't know

5.04 Please indicate whether you disagree with, agree with, or have mixed feelings about the following statements.

	**Disagree**	**Mixed feelings**	**Agree**
a. I/My colleagues don't have the support of the administration of my health facility to provide safe abortions.	☐	☐	☐
b. I feel that the people who provide Comprehensive Abortion Care counseling at my health facility encourage women to keep the pregnancy.	☐	☐	☐
c. In my facility some professionals treat women badly for seeking an abortion.	☐	☐	☐
d. In my facility, women seeking an abortion who are seen by a service provider who is opposed to abortion are never referred to another doctor.	☐	☐	☐
e. The supplies to perform abortions (e.g. misoprostol, MVA syringes) are usually or always available.	☐	☐	☐
f. My supervisor believes that abortions are morally wrong.	☐	☐	☐
g. Clinicians refusing to provide abortions because of moral or religious beliefs is one of the main barriers to women accessing safe, legal abortion.	☐	☐	☐

5.05 Do you know any clinicians who are trained in abortions, and who…

	Yes	No
Don't provide abortions because of their moral or religious beliefs	☐	☐
Refuse to provide abortions in one health facility, but provide them in another facility	☐	☐
Charge clients money besides what the facility charges, to provide abortion	☐	☐

Section 6: Possible policies

We are interested in your opinions about possible ways to regulate conscientious objection so that women can access legal abortion services, while abortion providers and conscientious objectors alike feel able to do their jobs in a fair environment. Some of the following regulations are used in other countries.

6.01 In your personal opinion, would the following regulations be a good idea to implement in Ghana?

Potential policy	No	Unsure	Yes
a. Mandatory **confidential** registration of conscientious objectors with GHS	☐	☐	☐
b. Mandatory **public** registration of conscientious objectors with GHS	☐	☐	☐
c. Mandatory **confidential** registration of conscientious objectors with the facility in which they work	☐	☐	☐
d. Additional compensation for providers who perform abortions	☐	☐	☐
e. Alternative service (for example, working additional hours at other clinical tasks) for providers who are conscientious objectors	☐	☐	☐
f. A penalty (for example, a monetary fine) for providers who are conscientious objectors	☐	☐	☐
g. A requirement by the Medical and Dental Council that OB/GYNs learn how to provide abortions	☐	☐	☐
h. A mandate that health facilities create and disseminate facility-level guidelines about conscientious objection	☐	☐	☐

6.02 In your personal opinion, how should the Ghana Health Service regulate the practice of conscientious objection?

____

____

____

6.03 In your personal opinion, what are some ways that the Ghana Health Service could encourage providers to perform abortions when needed?

____

____

____

Section 7: Religion

7.01What is your religion?

- ☐ Catholic
- ☐ Methodist
- ☐ Presbyterian
- ☐ Pentecostal/ Charismatic
- ☐ Other Christian. Specify___________________
- ☐ Muslim
- ☐ Traditional/ Spiritualist
- ☐ Hindu
- ☐ Pagan
- ☐ No religion
- ☐ Other (specify) ___________________

7.02 How much does your religion influence your everyday life?

- ☐ In few areas
- ☐ In many areas
- ☐ In everything I do

Section 8: Conclusion

8.01If you have other thoughts you would like to share about conscientious objection, legal abortion, or postabortion care, please add them here.

____

____

____

This concludes the survey. Thank you very much for your time and your responses.

TO BE COMPLETED BY GDC/GHANA

9.01 Is this facility urban or rural?

- ☐ Urban
- ☐ Rural

9.02 In which region is this facility located?

- ☐ Northern Region
- ☐ Upper West Region
- ☐ Upper North Region

9.03 What type of facility is this?

- ☐ Public
- ☐ Private
- ☐ CHAG
